# Hybrid Metamaterial Absorber Platform for Sensing of CO_2_ Gas at Mid‐IR

**DOI:** 10.1002/advs.201700581

**Published:** 2018-02-21

**Authors:** Dihan Hasan, Chengkuo Lee

**Affiliations:** ^1^ Department of Electrical and Computer Engineering National University of Singapore 4 Engineering Drive 3 Singapore 117576 Singapore; ^2^ NUS Suzhou Research Institute (NUSRI) Suzhou Industrial Park Suzhou 215123 P. R. China; ^3^ Center for Intelligent Sensors and MEMS National University of Singapore E6#05‐11F, 5 Engineering Drive 1 Singapore 117608 Singapore; ^4^ Graduate School for Integrative Science and Engineering National University of Singapore Singapore 117576 Singapore

**Keywords:** absorbers, complementary metal–oxide semiconductors, CO_2_, metamaterials, nondispersive infrared sensing, selective

## Abstract

Application of two major classes of CO_2_ gas sensors, i.e., electrochemical and nondispersive infrared is predominantly impeded by the poor selectivity and large optical interaction length, respectively. Here, a novel “**hybrid metamaterial**” absorber platform is presented by integrating the state‐of‐the‐art complementary metal–oxide–semiconductor compatible metamaterial with a smart, gas‐selective‐trapping polymer for highly selective and miniaturized optical sensing of CO_2_ gas in the 5–8 µm mid‐IR spectral window. The sensor offers a minimum of 40 ppm detection limit at ambient temperature on a small footprint (20 µm by 20 µm), fast response time (≈2 min), and low hysteresis. As a proof‐of‐concept, net absorption enhancement of 0.0282%/ppm and wavelength shift of 0.5319 nm ppm^−1^ are reported. Furthermore, the gas‐ selective smart polymer is found to enable dual‐mode multiplexed sensing for crosschecking and validation of gas concentration on a single platform. Additionally, unique sensing characteristics as determined by the operating wavelength and bandwidth are demonstrated. Also, large differential response of the metamaterial absorber platform for all‐optical monitoring is explored. The results will pave the way for a physical understanding of metamaterial‐based sensing when integrated with the mid‐IR detector for readout and extending the mid‐IR functionalities of selective polymers for the detection of technologically relevant gases.

## 1 Introduction

Increasing rate of environmental pollution is causing monumental and irreversible damage to the earth as the rapid urbanization and industrialization take place across the globe. Air pollution is currently regarded as the most harmful form of environmental pollution which is caused by a myriad of natural and manmade agents. Among them, emission of greenhouse gases (e.g., **CO_2_**) causing global warming has been a critical environmental concern over the last decade.^1^, ^2^ Apart from emission control, precision sensing of CO_2_ gas is indispensable for indoor air quality (IAQ) monitoring and heating, ventilation and air conditioning systems, industrial storage and refrigeration, and medical applications such as capnography. Many efforts have been invested for the development of application‐specific CO_2_ sensors. Two major approaches include (i) electrochemical sensing using gas‐selective ceramics^3^ and (ii) nondispersive infrared (NDIR) sensing using optical elements, i.e., microheaters, filters, and infrared sensors.^4^ Although, electrochemical sensors are widely used in market and could be ideally further downsizing for portable electronic applications, such sensors strongly suffer from poor selectivity and large power consumption and hysteresis. Current NDIR platform promises lower hysteresis, but selectivity is still limited in a mixed sensing environment where various pollutant gases including water vapor coexist interfering at the same operating wavelength. More importantly, NDIR sensors require centimeter long optical interaction length with low roughness sidewall in order to achieve ppm level detection limit with high signal‐to‐noise ratio (S/N ratio). Therefore, present NDIR devices are bulky and limited for personalized applications. Recently, 10 000 ppm detection limit is achieved in an NDIR setup with a ≈7.5 mm long optical interaction length, where it also reports characterization of metamaterial emitter.^5^ While metamaterial emitters for producing radiation of wavelength with high‐quality factor have been investigated by a few groups,^6^, ^7^ enhanced absorption characteristics in mid‐IR spectrum by using metamaterials absorber for gas detection with high selectivity has not been reported. In contrast with metamaterial emitter, metamaterial absorber enables the conversion of light into heat for read out by the integrated system. With the aid of metamaterials absorber, the gas sensing system can achieve high wavelength selectivity, polarization dependence, and controlled light‐matter interaction.^8^, ^9^, ^10^, ^11^, ^12^, ^13^, ^14^ However, due to the small size of CO_2_ molecule (232 pm), the near field coupling between the metamaterial absorber and gas is still limited. Besides, the all kind of surrounding gas molecules including water vapor will again contribute signature peaks and wavelength shift in this sensing platform when we apply it in practical. It means the selective detection of particular gas is not achievable in the metamaterial absorber‐based NDIR sensing platform. By incorporating a gas‐selective‐trapping polymer into the existing NDIR sensing platform, we can overcome this grand challenge of gas sensing.

Here, we combine two different sensing mechanisms, i.e., the infrared finger prints associated with the electrochemical response of a smart, gas‐selective‐trapping polymer and enhanced absorption characteristics of metamaterial absorber in the mid‐IR spectra, into a “hybrid device” as a new miniaturized optical gas sensor.^15^, ^16^ The miniaturization is realized as the gas‐selective‐trapping polymer layer physically captures and concentrates the CO_2_ molecules of the surrounding area. Near‐field coupling of the polymer with the metamaterial absorber enables the sensitive detection of chemical transformation of the polymer driven by the capture of gas molecules. The proposed approach exhibits ppm range of detection limit (40 ppm as reported in this work) implying at least 250 times better sensitivity than the previous work,^5^ while the optical interaction length (1 mm) is reduced by approximately seven times and shows high selectivity against humidity and various other pollutant gases. Hence, our new approach is a promising solution for future gas sensing systems. Here, we identify a suitable CO_2_ sensitive, amine‐based chemisorbent for the post‐complementary metal–oxide–semiconductor (CMOS) integration in order to realize a “**hybrid metamaterial**” absorber platform. Previously 2D materials such as graphene and MoS_2_ with exotic properties show selective gas sensing features particularly at a very low concentration.^17^ However, such approach is predominantly limited to electrical detection and does not work well in optical detection due to the weak optical response of 2D materials. Also, certain plasmonic metal (e.g., Pd) can selectively form chemical compound with reactive agent and particularly allows sensing of chemically active gases (e.g., H_2_).^18^ However, the abovementioned approach does not work for sensing the chemically inert gases such as CO_2_. Although porous metal–organic framework can facilitate the trapping of CO_2_ molecules, the infrared absorption intensity of the trapped molecules is typically low requiring long interaction length to generate readable signals above the noise level. Recently a detection limit of ≈60 000 ppm is demonstrated by Wang and co‐workers in a 5 mm long device based on such principle.^19^ Therefore, chemically active gas‐selective polymer such as amine‐based chemisorbent with rich mid‐IR functionalities has the potential for truly miniaturizing the CO_2_ gas sensor with ppm range of detection limit. The CMOS platform further promises scalable and repeatable integrated sensor manufactured at a very low cost. One critical challenge behind the integration is the compatibility between the CMOS compatible metal surface (Mo in this work) and gas‐selective thin film. Here, we report stable sensing characteristics of the platform implying strong adhesion between the two heterogonous layers for repeatable applications. Furthermore, an added advantage of the gas‐selective polymer for dual‐mode sensing is experimentally confirmed. Besides, we experimentally report unique sensing characteristics of the platform at various ranges of gas concentration and the optimized condition for maximum differential response. This work will primarily focus on the detailed study of the proposed versatile platform from its optical physics and chemical functionalities' point of view. We believe, the findings will lay the solid foundation for the successful system‐level integration of the platform in future.

## 2 Hybrid Metamaterial Absorber Platform

**Figure 1:**
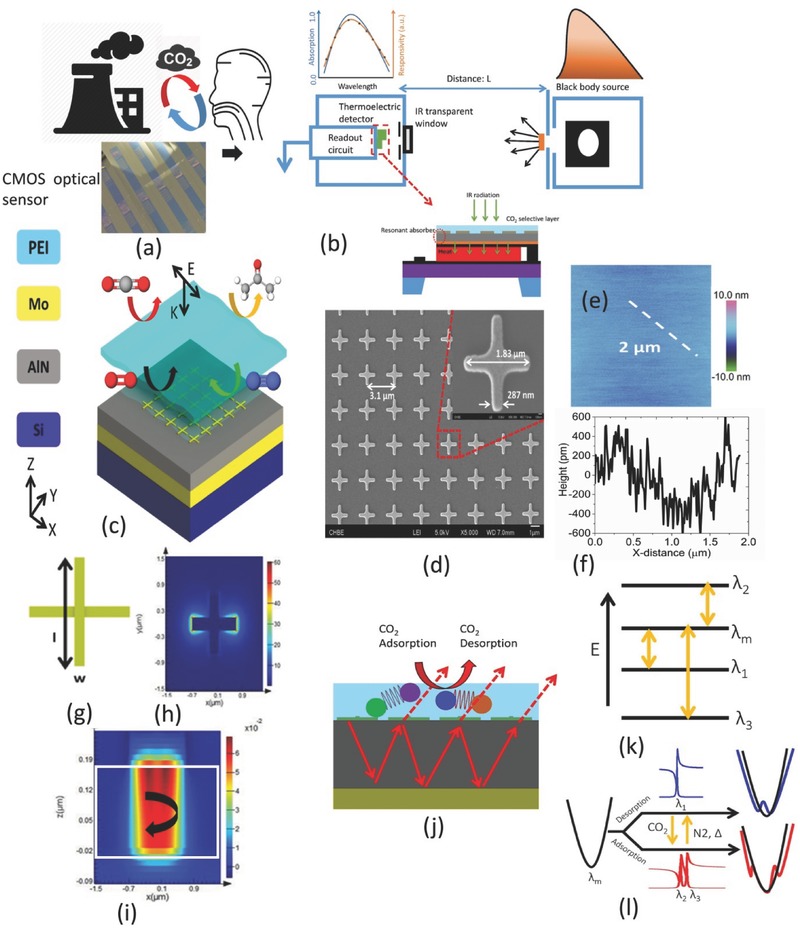
a) Increasing emission of CO_2_ as one of the emerging environmental risk factors for living beings demanding large‐scale deployment of sensors achievable only under low cost, CMOS platform. b) Conceptual diagram of state‐of‐the‐art nondispersive infrared (NDIR) sensing platform. “Active metamaterial” absorber platform with the potential for physically trapping CO_2_ gas and thereby, reducing the gas cell length for miniaturized sensor is indicated accordingly. c) Proposed “hybrid metamaterial” absorber integrated with thin film membrane for selective gas sensing. d) FESEM (field emission scanning electron microscopy) image of the array of the metamaterial patterns (Inset: zoomed in view of the unit cell). e) AFM image of the PEI surface coated on the metamaterial patterns. f) AFM height profile of the PEI surface. g) Crosswire metamaterial pattern for polarization independent sensing characteristics h). i) 3D FDTD (finite‐difference time‐domain) simulated electric field and magnetic field intensity distribution along *XY* plane and *XZ* plane of the absorber device, respectively, at the perfect absorber resonant condition. Confinement of H‐field in the dielectric spacer region leading to antiparallel current flow between the top metal and bottom metal at absorption resonance is marked by the white rectangular boundary. j) Coupling of metamaterial absorption resonance mediated by thin film interference into the vibrational modes of CO_2_ selective layer for the sensing (adsorption and desorption) k). l) Energy diagram representation and effect of coupling to closely spaced material vibrations on metamaterial resonance, respectively. Presence of infrared material vibrations is strongly subject to gas adsorption and desorption providing multiple routes for the selective detection of CO_2_ gas.

**Figure** **1** represents the mid‐IR metamaterial platform for CO_2_ sensing application. The emerging issue of greenhouse CO_2_ emission is illustrated in Figure 1a for which massively deployable, CMOS compatible sensors are necessary. Conceptual integration of the hybrid metamaterial platform for CO_2_ sensing with the state‐of‐the‐art NDIR system (Figure 1b) is presented in Figure 1c.^20^ Figure 1c shows the post‐CMOS integration of gas‐selective, enrichment layer (polyethylenimine, PEI). The representative metamaterial pattern in this work is shown in Figure 1d. AFM (atomic force microscopy) image in Figure 1e and roughness plot in Figure 1f strongly suggest the uniformity of the spin‐coated PEI film on metamaterial patterns. The metamaterial absorber is consist of metal‐dielectric spacer‐metal layers. The dimensional parameters (length: *l* and width: *w*) of the metal patterns at the top layer are shown in Figure 1g. In this work, the width of the pattern is kept fixed at ≈287 nm being limited by the critical dimension of the lithography process. The thicknesses of the top metal layer, dielectric spacer, and bottom metal layer are fixed at 100, 200, and 200 nm, respectively. The resonant E‐field distribution at the device plane (*XY* plane) and H‐field distribution at the device cross‐section are given in Figure 1h,i, respectively, in which a plane wave light source is incident on the 3D structure from the top. The polarization of the incoming radiation is fixed along *x*‐axis while the probe wavelength is set to be 6.5 µm.

In particular, integration of metamaterial platform with such gas‐selective smart material offers two specific features to improve the performance of the gas sensor. First, it can overcome the weak near‐field interaction between gas molecule in free space and plasmonic metamaterial pattern.^21^ Second, it not only enables the effective utilization of the rich physics of metamaterials for gas sensing applications but also provides a scalable route for optical sensing of any gas on demand depending on the wavelength characteristics of the selective membrane. For example, coupling with the slow light has been reported to enhance the infrared absorption intensity by at least 1000 times ultimately improving the linearity and range of detection of the hybrid optical sensor.^22^ In general, gas sensing at infrared is based on Beer–Lambert law: *I* = *I*_0_ exp (−*γ α L*) where *I* is the transmitted light intensity and *I*_0_ is the incident intensity, α is the effective absorption coefficient of the gas, which is determined by the gas concentration and is strongly dispersive. *L* is the interaction length and γ is the enhancement factor determined by the sensor structure. In state‐of‐the‐art NDIR system without any gas‐selective enrichment layer, γ can be assumed to be equal to 1. Here, we propose that γ can be greater than 1 reducing the overall interaction length for sensing through the near‐field coupling of metamaterial patterns with the infrared absorption bands of the gas specific chemical species within the gas‐selective layer. Perfect absorption condition as the reference can be further ensured in the hybrid system through metamaterial design principles for sensor operation above the noise margin in the integrated system.^23^, ^24^ The heat convection loss in the integrated system can be further overcome by the matured technology of chip‐scale vacuum encapsulation.^25^ Besides, we report the ultimate advantages of strengthening infrared absorption of the gas‐selective polymer by surface enhancement via near field coupling so as to maximize the overall sensitivity.^26^ Additionally, the advantage of metamaterial absorber for reflection‐based all‐optical monitoring with large differential response is explored.

Figure 1j shows the coupling of interference in thin film spacer with the gas‐selective layer. Due to the presence of multiple, chemically active vibrational modes, such coupling is unique for CO_2_ adsorption and desorption event. The energy level diagram showing the multiple paths of coupling of metamaterial mode (λ_m_) into the adjacent, closely spaced (≈250–500 nm in this work) vibrational modes (λ_1_, λ_2_, and λ_3_) for sensing applications is shown in Figure 1k. Again, the quality factor of metamaterial absorbers at mid‐IR is typically in the range of 5–10 at the corresponding 5–8 µm spectral region and therefore can span over the closely spaced vibrational finger‐prints. CO_2_‐dependent coupling is illustrated in Figure 1l exhibiting the effective broadening of the background metamaterial mode.

## 3 Integration of CO_2_ Selective Polymer with Metamaterial Absorber

### 3.1 Material Characterization for CO_2_ Sensing

**Figure 2:**
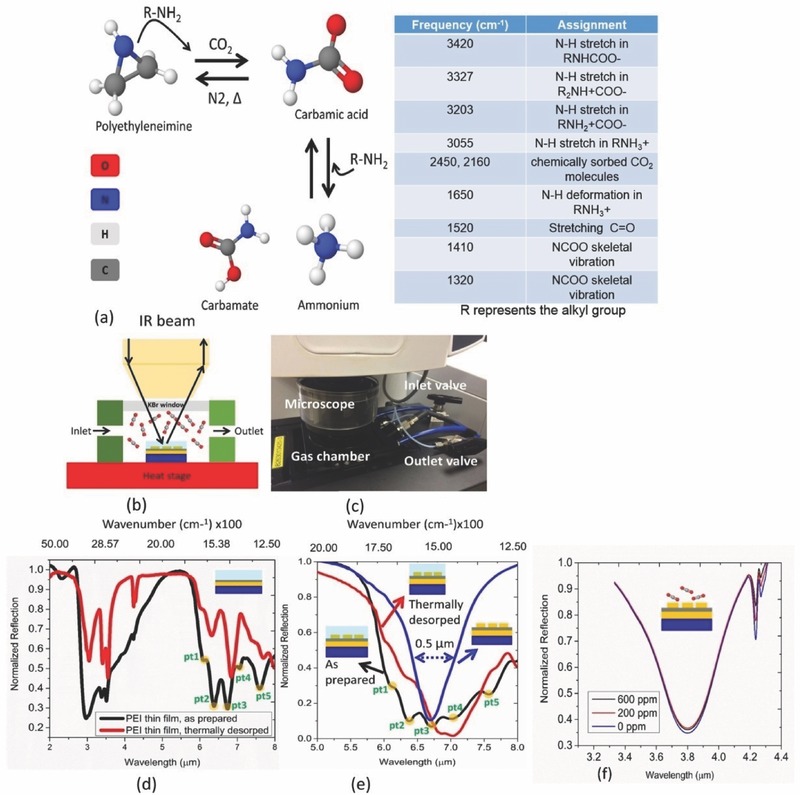
a) Chemical reactions followed by the adsorption and desorption of the CO_2_ sensitive layer and frequency assignments for the possible byproducts of the reaction path. Upon heating in a nitrogen environment, the formed species can recover back to original polyetheleneimine with negligible loss. b) Schematic of the gas sensing setup and c) gas sensing setup used for the experiment. The volume of the gas chamber used is 100 cm^3^. Gas flow rate is maintained at 500 mL min^−1^ with N_2_ used as the carrier gas. d) Comparison between as prepared (exposed to 600 ppm CO_2_ in ambient) and thermally desorped (treated at 100 °C for 2 min) PEI film. e) Effects of as prepared and thermally desorped PEI film on the resonance profile of the crosswire absorber structure. Five distinct frequency points are identified (pt1: 6.06 µm, pt2: 6.57 µm, pt3: 6.75 µm, pt4: 7.092 µm, and pt5: 7.57 µm) for studying the gas sensing characteristics of the hybrid geometry. f) Response of the metamaterial absorber structure in the absence of gas‐selective layer as exposed to various CO_2_ concentrations at 70% ambient humidity.

The gas selective behavior of the enrichment material can be optically probed in details in the mid‐IR spectrum. The CO_2_ selectivity is achieved due to its covalent interaction with the amine rich polymer. The chemical reactions involved with the adsorption and desorption of CO_2_ in the active layer and the associated frequencies are described in **Figure** **2**a. Clearly, mid‐IR spectroscopy opens up the possibility of implementing sensors with multipath selectivity and sensitivity out of the smart material. Detailed chemical reactions involved as CO_2_ reacts with the primary and secondary amines of the branched PEI polymer chain are as below

(1) $${\mathrm{CO}}_{2}\;\;+\;\;{\mathrm{2RNH}}_{2}\;\;=>\;\;{\mathrm{RNHCOO}}^{-}\;+\;\;{\mathrm{RNH}}_{3}^{+}$$

(2) $${\mathrm{CO}}_{2}\;\;+\;\;{\mathrm{2R}}_{2}\mathrm{NH}\;\;=>\;\;R_{2}{\mathrm{NCOO}}^{-}\;+\;\;R_{2}{\mathrm{NH}}_{2}^{+}$$

(3) $$\begin{array}{l} {\mathrm{CO}}_{2}\;\;+\;\;R_{2}\mathrm{NH}\;\;+\;\;R^{\prime }{\mathrm{NH}}_{2}\;\;=>\;\;R_{2}{\mathrm{NCOO}}^{-}\;+\;\;R^{\prime }{\mathrm{NH}}_{3}^{+}\\ \;\;\;\;\;\;\;\;\;\;\;\;(\mathrm{or}\;R^{\prime }{\mathrm{NHCOO}}^{-}+\;\;R_{2}{\mathrm{NH}}_{2}^{+}) \end{array}$$

In the presence of humidity, an equimolar reaction takes place as below

(4) $$R_{2}\mathrm{NH}\;\;+\;\;{\mathrm{CO}}_{2}\;\;+\;\;H_{2}O\;\;=>\;\;R_{2}{\mathrm{NH}}_{2}^{+}\;\;+\;\;{\mathrm{HCO}}_{3}^{-}$$

We further perform the electrical charge characterization of the smart material under various CO_2_ conditions and confirm the selectivity behavior of the obtained thin film (Figure S1, Supporting Information). The measured electrical results strongly correlate with the formation of infrared species with unique fingerprints at the surface of the PEI gas‐selective layer.

In this work, we particularly focus on the infrared bands located in the range from 1300 cm^−1^ (7.692 µm) to 1700 cm^−1^ (5.882 µm) for the sensor calibration at steady state. Interestingly, the chemical fingerprints around 3055–3420 cm^−1^ show unusual self‐recovery as the sampling gas leaves the chamber by diffusion (Figure S2, Supporting Information).

### 3.2 Proof‐of‐Concept Demonstration of CO_2_ Sensing at Steady State

A bare 8″ silicon wafer was cleaned and 200 nm of Mo followed by the AlN thin film for the spacer and the top 100 nm Mo film for the patterns were sputtered under high vacuum. Deep UV photolithography process was used to define the metamaterial patterns. Finally, Mo was dry etched to form the final absorber structure. The gas‐selective layer is obtained by spin coating at various speeds. A maximum thickness of 300 nm was obtained from the prepared solution at a speed of 1000 rpm. For characterization, high‐resolution Fourier transformed infrared (FTIR) microscope integrated with a heat stage and sealed gas cell chamber was deployed. The reflected signal is normalized with respect to a smooth gold surface. The incident light source is unpolarized unless otherwise stated. The integrated setup is shown in Figure 2b,c. The calibration of the sensor is confirmed by a commercial CO_2_ meter. The properties of the materials chosen for the hybrid sensor are listed in **Table** **1**. Mechanical matching is obtained between AlN and Mo on purpose to minimize the hysteresis effect due to thermal stress over repeat cycles of thermal reset (desorption). Besides, high thermal conductivity of AlN is considered in order to ensure fast heat transfer between the absorber and the detector in the integrated system.^27^

**Table 1:** Material properties of the hybrid platform

Material	Melting point [°C]	Young's modulus (*E* [GPa])	Poisson ratio [µ]	Coefficient of thermal expansion: CTE (× 10^–6^ K^–1^)	Thermal conductivity [W m^−1^ k^−1^]
Aluminum nitride	2200	344.8	0.287	4.6	285
Molybdenum	2620	329	0.32	4.8	140
PEI	95	2.9	0.44	56	0.23

Figure 2d shows the behavior of the PEI film on a metallic substrate without any pattern as it is exposed to ambient CO_2_ sorption and thermal desorption. A strong contrast of intensity (60%) is observed at around 3 µm due to the formation of the unique infrared chemical species by the exposure to CO_2_. On the other hand, five distinct spectral points are identified within the range 5–8 µm which we leverage in this work for the sensor calibration. Figure 2e is the proof‐of‐concept demonstration of the sensing behavior of the hybrid platform on a footprint of 20 µm by 20 µm. The length (*l*) of the corresponding pattern is 2.3 µm yielding maximal overlap with the closely spaced vibrational modes. The pristine device almost maintains perfect absorption condition (*A* = 1 − *R*) by design. The perfect absorption condition still prevails in its hybrid form in both conditions, i.e., exposed to CO_2_ and thermally desorped. Notably, microheater‐based thermal swing integrated with metal‐based metamaterial platform is achievable here for fully on‐chip reset of the sensor via thermal desorption. The observed concentration‐dependent modulation of the net structural absorption can be further correlated with the light‐to‐heat conversion in the integrated system. In contrast with the hybrid structure, the uncoated metamaterial absorber shows negligible response toward the exposure of CO_2_ in Figure 2f. The refractive index (RI) induced shift can be calculated as Δ λ   =   m(nCO2−  nN2​)(1  −  e−2d/ld​​​) where *m* is the RI sensitivity [in nm/RI unit (RIU)], *d* is the effective thickness of medium and *l*_d_ is the electromagnetic field decay length of the metamaterial patterns in the order of few hundred nanometer.^28^ In the case without the gas‐selective layer, *d* >> *l*_d_, while the refractive indices of CO_2_ and N_2_ are 1.0004 and 1.0002, respectively. The average RI sensitivity of the metamaterial absorber structures are experimentally found to be 500 nm/RI unit. Therefore, the maximum wavelength shift expected is 0.1 nm while the observed shift in Figure 2f is ≈1 nm. The additional shift can be attributed to the adsorption of water vapor (water contents: ≈20 ppm v/v in the food grade CO_2_) by the lightly oxidized Mo surface at the given inlet pressure of the miniaturized gas chamber in this work.^29^ Although a strong correlation between the intensity of the CO_2_ finger print and gas concentration is observed in Figure 2f, no such definite trend can be observed at around the resonance wavelength of the structure clearly implying the random nature of the physical adsorption of foreign molecules. On the other hand, the active device proposed in this work shows high sensitivity to CO_2_ concentration under the identical sensing condition.

It is notable that a broadening phenomenon is observed when the background metamaterial is coupled to the group of vibrational modes in both cases. Under thin film assumption, such broadening can be contributed by the recurrent mode splitting as expressed by the following equations^30^, ^31^

(5) $$E_{\pm }=\frac{E_{m}+E_{\mathrm{vib}}}{2}\pm \sqrt{\frac{{\left(E_{m}-E_{\mathrm{vib}}\right)}^{2}}{4}}+\nu^{2}$$

**Figure 3:**
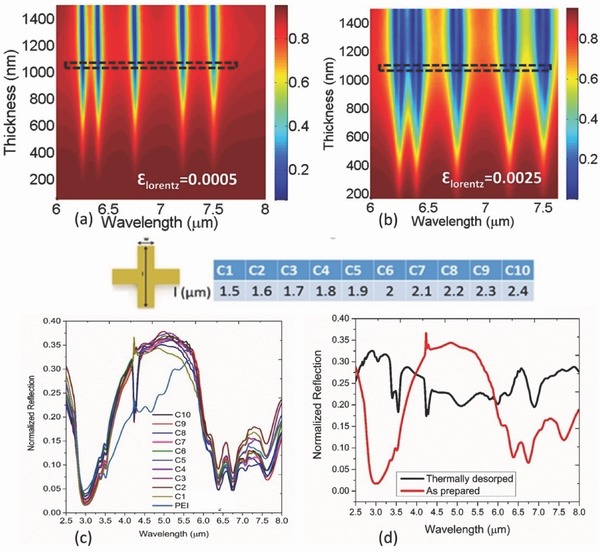
Thickness versus wavelength map of the reflection spectrum of the polymer‐metal geometry. a) Oscillator strength ε_lorentz_ fixed at 0.0005. b) Oscillator strength ε_lorentz_ fixed at 0.0025. c) Length dependence of the reflection spectra of the metamaterial patterns (C1–C10) coated with 300 nm PEI film (as prepared, i.e., exposed to ambient CO_2_) when the back metal reflector is removed. d) Reflection spectra of the similar C1 structure coated with 300 nm PEI film at two different states: (i) as prepared (ii) thermally desorped showing no strong coupling with the background resonance of the pattern.

Here, *E*_m_ and *E*_vib_ are the energy state of the background metamaterial mode and material vibrational mode, respectively, and ν is the coupling strength between the modes. Such coupling can also lead to hybrid features that are spectrally shifted from the isolated vibrational modes. However, in the thick film case for sensor applications, we also take into account the influence caused by the vibrational modes on the destructive interference condition between the direct reflection from the air–device interface and the subsequent reflections as shown in Figure 1g.^32^ Although, in absorber case, the superimposed reflection defined as $\tilde{R}\;\;=\tilde{r}\;\;+{\tilde{r}}_{1}\;\;+{\tilde{r}}_{2}\;\;+{\tilde{r}}_{3}\;\;+\;\;\cdots$ can be minimized at a single wavelength for a given mode, additional decrease of reflection at adjacent wavelengths can be still expected due to the multiple interactions within the absorbing overlayer before the light escapes. Such phenomenon can further aid the broadening behavior and is strongly supported by the results in Figure 2. To investigate the effect, we analytically derive the reflection of the gas‐selective polymer assumed to be deposited on a metallic substrate. The overall reflection governed by the thin film interference can be defined as $\tilde{r}\;\;=\;\;[{\tilde{r}}_{12}\;\;+\;\;{\tilde{r}}_{23}\exp(2i\tilde{\beta })]/[1\;\;+\;\;{\tilde{r}}_{12}{\tilde{r}}_{23}\exp(2i\tilde{\beta })]$ where the subscripts 1, 2, and 3 denote the air, dielectric polymer, and metal region, respectively, and β implies the propagation constant in the dielectric region. The gas‐selective material is modeled by five Lorentz oscillators. The Fano‐type resonances are defined by ε​ =​ εo+∑i=15εlorentzωi2/(ωi2−2iδoω−ω2) where ε_o_ = 2.2500 and δ_o_ is fixed at 8 × 10^11^ rad s^−1^, respectively. Two different oscillator strengths (ε_lorentz_): 0.005 and 0.025 have been considered. The spectral positions of the five oscillators are fixed at (i) 6.25 µm, (j) 6.4 µm, (k) 6.75 µm, (l) 7.20 µm, and (m) 7.5 µm. **Figure** **3**a,b shows the thickness dependence of the interference controlled reflection off the structure at ε_lorentz_ = 0.005 and ε_lorentz_ = 0.025, respectively. Clearly, a saturation of reflection dip is observed in both the cases as the thickness increases (marked by dashed black rectangles). In this particular case, the spectral position and intensity of the reflection dips are strongly determined by the extinction coefficients (*k*) of the lossy dielectric. It is obvious that the destructive interference condition supported by the back metal reflector in the presence of loss (*k*) can completely suppress the reflection and achieve perfect absorption. Complete suppression of reflection is otherwise not found to be achievable in two‐layer geometry in the absence of the wavelength selective loss in the dielectric layer. Subsequently, such enhanced interaction can lead to strong overlap of the absorption bands for a given line width of the bands and cause effective broadening of the dips. Figure 3b depicts the phenomenon as the strength of the oscillators is scaled up five times. Metamaterial absorber geometry properly designed to cover the spectral locations of the available phonon modes of the polymer can therefore effectively enhance the absorption that ultimately leads to the broadening of the underlying metamaterial absorber mode. The role of thin film interference supported by the back metal reflector can be further verified by the experimental results in Figure 3c,d, where the back metal reflector is removed on purpose while the thickness of the top PEI film is kept constant at 300 nm. The results as a function of the length (*l*) of the metamaterial pattern show no strong evidence of coupling with the underlying resonance and closely correspond to the characteristics of PEI film alone. It implies significant screening of the weakly resonant patterns by the 300 nm film in the absence of back metal reflector. Therefore, metamaterial absorber structure considered in this work not only provides the wavelength selectivity but also ensures strong interaction with the sufficiently thick gas‐selective film as needed for the sensing application.

We further conduct a representative simulation of the metamaterial absorber structures using 3D finite‐difference time‐domain (FDTD) method. The results also correspond to the overlapping of modes that ultimately leads to such broadening (Figure S3a–f, Supporting Information). It is also observed that the coupling into all of the modes reaches saturation at a particular thickness (Figure S3g, Supporting Information) while a given mode can reach saturation at lower thickness depending on the spectral location of the background metamaterial mode. Also, the field intensity distributions in Figure S3i–n in the Supporting Information strongly indicate the magnetic component of the hybrid modes with the potential for efficient light‐to‐heat conversion for further integration. Finally, the role of absorption resonance of the metamaterial pattern on the coupling with closely spaced vibration modes can be confirmed by the simulated comparison as the metamaterial pattern is absent in Figure S4 in the Supporting Information.

The uniformity of the characteristics of the hybrid device over a large area is confirmed by the AFM imaging (Figure S5, Supporting Information) and focal plane array (FPA) imaging (Figure S6, Supporting Information). Again, the thickness‐dependent spectra in Figure S5a,b in the Supporting Information show a good control over the post‐CMOS process. Transient data for thermal desorption further indicates the required time to be around 2 min for complete reset (Figure S7, Supporting Information). In the later section, we analyze the dynamic response of the hybrid sensor at the five selected spectral points.

### 3.3 Geometry‐Dependent Characteristics of the Sensing Platform and Its On–Off Switching Behavior in the Differential Spectrum

In this section, we experimentally study the geometry‐dependent characteristics of the metamaterial absorber sensor by varying the length of the crosswire geometry. The perfect absorption condition is satisfied only when the effective impedance defined as $z_{\mathrm{eff}}\;\;=\;\;\sqrt{\mu {\;}_{\mathrm{eff}}/\varepsilon_{\;\mathrm{eff}}}$ becomes equal to the input impedance (*z*_in_) and the electrical (dipolar) and magnetic resonance simultaneously lead to the matching condition. However, in the presence of gas‐selective top layer with multiple vibrational modes, it is challenging to achieve perfect absorption condition across the whole spectrum without changing the spacer thickness. In particular, perfect absorption condition (*R* = 1 − *A*) is required to achieve near infinity differential response (*R*_sens_/*R*_o_ − 1) for sensing framework operating in the reflection mode where *R*_sens_ is the reflection intensity at the exposure of CO_2_ and *R*_o_ is the reflection intensity in the pure state. Differential response with high switching contrast is favorable for fiber‐based all‐optical sensing of gas within a miniaturized package. More importantly, perfect absorption condition assures maximum sensitivity of the hybrid platform when it is integrated with the NDIR system as shown in Figure 1b by enabling its operation above the noise margin of the thermoelectric transducer.

**Figure 4:**
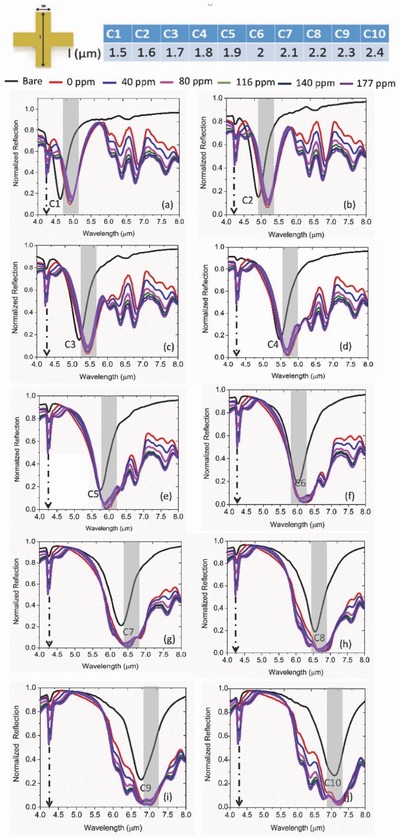
Geometry‐dependent gas sensing characteristics of the hybrid absorber structures a–j) C1–C10 geometry, respectively. The dashed arrow line locates the spectral position of CO_2_ molecule trapped inside the PEI overlayer. The gray region indicates the spectral position of the metamaterial absorption resonance in the background. Clearly, a significant spectral broadening is observed (e.g., C9) as the absorption resonance overlaps with the densely positioned vibrational modes of the gas sensitive layer. Also, the broadening increases as the gas concentration increases. Obvious redshift is observed as well (C1) as the gas concentration increases.

**Figure 5:**
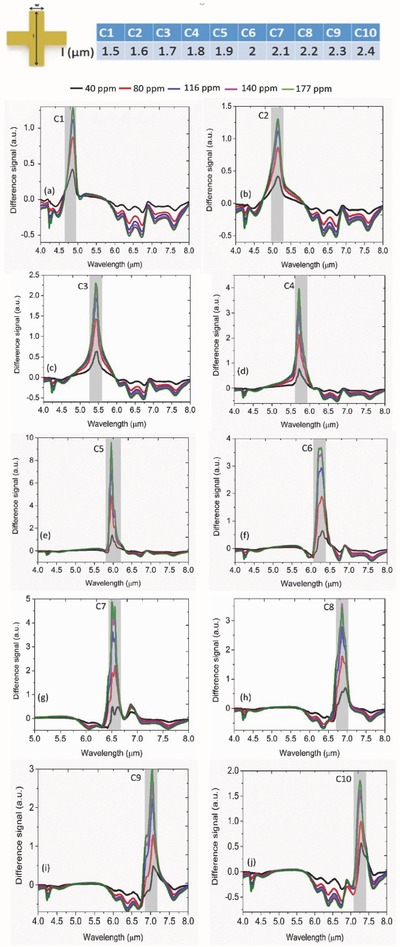
Geometry dependence of on–off switching observed in the differential spectra of the perfect absorber structures as a function of gas concentration a–j) C1–C10 geometry, respectively. The gray region indicates the spectral location of the metamaterial absorption resonance in each case. The peak switching contrast is observed to be in C5 geometry (≈9.85) as an outcome of the perfect impedance matching obtained in the hybrid geometry for a given thickness of the gas‐selective layer (≈300 nm). A pronounced splitting of the switching peak is observed (C6 and C7) that results from the interplay between the coupling of closely spaced vibrational modes and the background metamaterial absorption resonance. In all cases, the switching contrast scales up with the increased gas concentration.

Here, we scan across a broad spectrum (4–8 µm) by sweeping the length (*l*) of the geometry for the study. For calibration, we increase the CO_2_ concentration in continuous mode without any thermal reset and measure at steady state. As seen in **Figure** **4**a, a regular redshift is observed only for the C1 device. Such shift is directly attributed to the broadband intensity difference that is observed between 2.5 and 5 µm in Figure 2d due to the formation of unique chemical species in the presence of CO_2_. Note that the spectral position of C1 resonance is 4.9 µm that falls within the aforementioned range. Here, we report a wavelength shift 0.5319 nm ppm^−1^ calculated from the difference between 0 and 40 ppm. Apart from the resonance shift, an obvious intensity change as a function of CO_2_ concentration is observed in all the devices (C1–C5). The sensor, however, shows saturation behavior in continuous mode with no thermal reset which will be discussed in the following sections. Interestingly, this perfect absorption condition can be only achievable in the C5 device as seen from Figure 4e. Of course, the physics become slightly different as the background metamaterial mode approaches (C6 and beyond) the group of vibrational modes under current study. As stated before, significant broadening of the absorption band is clearly observed for such cases. We consider the hybridization to be maximum in the C9 device and explore it further for dynamic characterization. From quality factor's point of view, the C9 structure with its full width at half maximum of ≈1300 nm can maximally overlap with the five absorption modes located within a spectral range of 1500 nm. As a direct consequence of the perfect absorber condition, the on–off switching raio in the differential spectra reaches a maximum of 9.85 (a.u) in the C5 device (**Figure** **5**e) which again falls to 3.6 in the subsequent C6 device as the perfect absorption condition is interrupted (Figure 5f). Such high contrast in differntial reposne is particularly suitable for optical gas sensing at low concentration and in reflection mode. Additionally, a clear splitting is observed in the difference spectra (C6, C7 in Figure 5f,g, respectively) that we attribute to the strong overlap between the metamaterial mode and one of the vibrational modes. Again, the switching contrast reaches a maximum of 4.9 (C7) in this regime by satisfying the perfect absorption condition to a maximal degree.

### 3.4 Multiplexed Sensing at Steady State by the Proposed Platform

**Figure 6:**
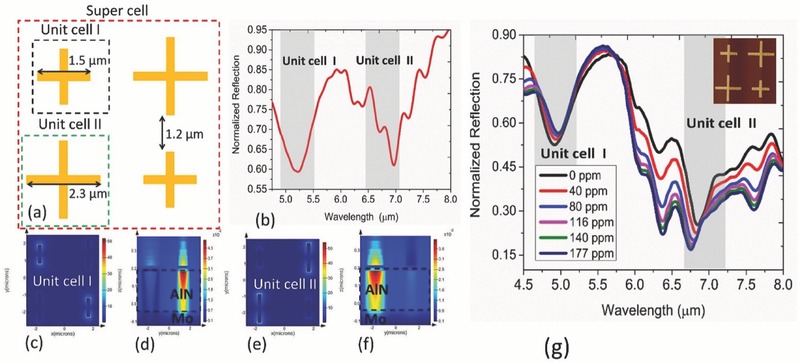
Demonstration of multiplexed sensing on the proposed platform at steady state condition a) design of super cell composed of unit cell I and unit cell II for multiplexed sensor b). Simulated resonance profile of the supercell geometry overlaid by the gas‐selective layer. Polarization of the incoming radiation is fixed along *y*‐axis c). d) Electric field (at *XY* plane) and magnetic field (at *XZ* plane) distribution, respectively, when unit cell I is resonant at 5.25 µm e). f) Electric field (at *xy* plane) and magnetic field (at *xz* plane) distribution, respectively, when unit cell II is resonant at 6.75 µm. g) Steady‐state sensing characteristics of the multiplexed platform. Inset: fabricated superpixel.

Multiplexed dual mode sensing is getting popular for NDIR technology where one wavelength is used for referencing the intensity of the sensor and another wavelength for the calibration of gas concentration.^33^ Based on the experimental results in the previous section, here we report a new multiplexing technique incorporating the signal variation in the coupled and uncoupled regimes. The supercell structure as shown in **Figure** **6**a is consist of two different unit cells, where one is designed to operate in the uncoupled regime and the other is designed for the coupled regime. We believe the potential of the gas‐selective material can be fully extracted through this manner by capturing both the refractive index‐dependent wavelength shift and coupling variation into the vibrational modes for dual mode sensing. The representative simulation result showing the resonance characteristics of the supercell in the presence of the hypothetical gas‐selective polymer is presented in Figure 6b. The respective resonance field profiles in Figure 6c–f show no sign of near‐field interaction within the unit cells. Figure 6g shows the proof‐of‐concept experimental results for the multiplexed sensing. At the unit cell I region, a monotonic wavelength shift is observed while at the unit cell II region, mainly a strong intensity variation is observed. In particular, superposition of resonantly (unit cell II) and nonresonantly (unit cell I) enhanced absorption bands at pt3 and pt4 spectral locations is noted. Overall, the concentration‐dependent change observed at the two regions indicates the multifunctional characteristics of the gas‐selective material suited for metamaterial‐based optical sensing. One fundamental limitation of the scheme observed is the side band overlap of the unit cell specific resonances that ultimately leads to large reflection in the background. For example, the minimum reflection observed around unicell I resonance is 52.5% meaning 47.5% effective absorption. Such limitation can be possibly overcome by designing high Q nanostrcture ensuring minimal spectral overlap between the sidebands.^34^

### 3.5 Dynamic Sensing Characteristics and Selectivity

#### 3.5.1 Dynamic Behavior of Average Absorption

**Figure 7:**
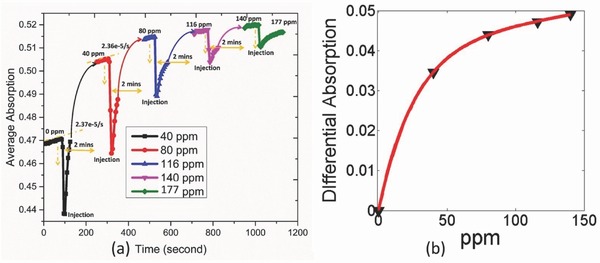
a) Dynamic behavior of average absorption in C9 geometry obtained by integrating the normalized absorption within the spectral window: 5–8 µm. b) Differential absorption at steady state fitted under 2 parameter exponential model (*  f*(*x*) = a*exp(b*x) + c*exp(d*x)) indicating saturation behavior of the sensor as the gas concentration increases in continuous mode. The fitting values of *a*, *b*, *c*, and *d* are 0.04555, 0.0004665, −0.04551, and −0.0691, respectively, with *R*‐square value of 0.9993.

In this section, we analyze the dynamic behavior of the hybrid sensor considering the average absorption calculated as $A_{\mathrm{avg}}=1/(\lambda_{2}-\lambda_{2})\int_{\lambda_{1}}^{\lambda_{2}}(1-R)d\lambda$, where λ_1_ and λ_2_ are the beginning and end wavelength of the averaging interval, respectively. Time‐dependent average absorption extracted in 5–8 µm range is provided in **Figure** **7**a that accounts for the variation of the all the vibrational modes. The abrupt decrease in the intensity at every instance of gas injection is due to the carrier N_2_ induced partial desorption and cleaning of CO_2_ induced infrared species. At every injection, the outlet is kept open so as to drive away the previous concentration from the chamber completely while the steady state measurement is taken keeping the outlet closed and ensuring the uninterrupted exposure of the sensor to a given concentration at zero flow rate. This mimics the real‐life situation in indoor air quality monitoring and avoids the N_2_ flow rate induced partial desorption at steady state. The steady state response therefore indicates the maximum signal output of the sensor in response to a particular concentration. The results indicate ppm level limit of detection with a response time close to 2 min. The slope indicated (2.36 × 10^−5^ s^−1^) implies the steady state condition achieved at various concentrations. The differential absorption calculated from the difference of two steady state conditions in Figure 7b shows the potential of the platform for sensing in continuous mode. However, saturation behavior of the sensor is still observed which we attribute to the limited capacity of CO_2_ storage within the thin film and can be improved by increasing the thickness of the film and prolonged desorption under N_2_ environment before each measurement. It is notable that the sensor triggers as soon as its exposed to a minute CO_2_ concentration (40 ppm in this work) making the platform highly senstivite to the prescence of the trace gas. We believe it is the adsorption of CO_2_ at the upper surface followed by the formation of infrared species that leads to such characteristics through the subsequent electromagnetic coupling. Furtheremore, the abrupt intensity change is self‐powered and therefore can be considered for futuristic, event‐driven air flow monitoring system.^35^ Besides, with the potential for integration with read‐out electronics, it is possible to perform the sensing on the rising edge even before reaching the steady state, and thereby reduce the overall power consumption.

**Figure 8:**
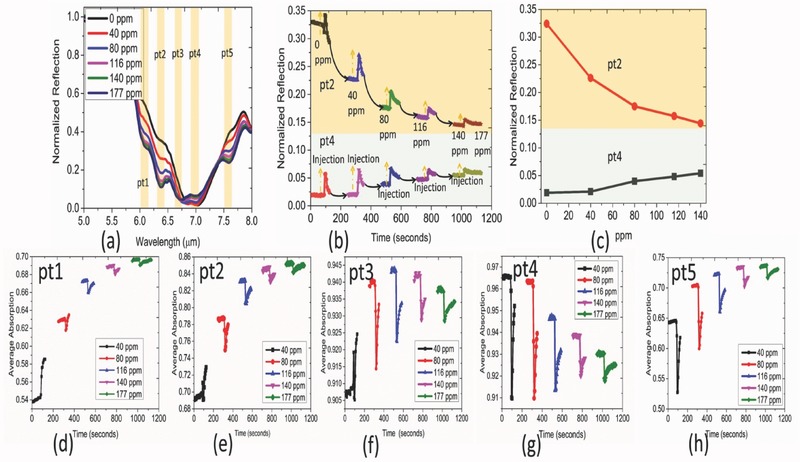
a) Steady‐state normalized reflection of C9 absorber structure at different concentrations. b) Time evolution of normalized reflection at two distinct spectral regions: pt2 and pt4 as the concentration increases in continuous mode. c) Extracted steady state normalized reflection. d–h) Time‐dependent average absorption as a function of gas concentration integrated over a bandwidth of 400 nm around pt1, pt2, pt3, pt4, and pt5 spectral position, respectively. Increasing trend is observed for pt1, pt2, and pt5 whereas decreasing trend is observed for pt4. Average absorption is found to reach a maximum at intermediate concentration (80 ppm) at pt3 location.

Later, we breakdown the average absorption into five exact bands (400 nm each) around the spectral points (pt1, pt2, pt3, pt4, and pt5) and analyze the contribution from each under resonant approxmiation. Clearly, all the bands except to pt3 show the sensing behavior. The normalized reflection spectra extracted at pt2 and pt4 center wavelength in **Figure** **8**b are also in harmony with the trend of the calculated, band‐specific average absorption in Figure 8e,g. It is worthwhile to note that the saturation characteristics is much improved in the case of pt4 (7.092 µm) band that corresponds to NCOO skeletal vibration indicating the higher capacity of this reaction path for CO_2_ sensing. The absence of any monotonic trend in the case of pt3 refers to the intricate dependence of the coupling on the gas concentration. As the concentration builds up, a crossing behavior becomes clearly observable in Figure 8a that leads to a nonmonotonic trend at this spectral location. The dynamic behaviors are also collected at two different nonresonant wavelengths (Figure S8, Supporting Information). Although the sensing behavior is observed by the trapped CO_2_ molecules (4.23 µm), no particular trend is observed at 5.6 µm at 116 ppm and beyond. This is because of the crossing among the spectra which is previously observed in Figure 4i as the concentration builds up. The results, therefore, indicate the need for a judicious selection of the wavelength range to ensure reliable sensing on the proposed platform.

#### 3.5.2 Dynamic Sensing Beyond Safe Level, Ambient CO_2_ Concentration and Selectivity

**Figure 9:**
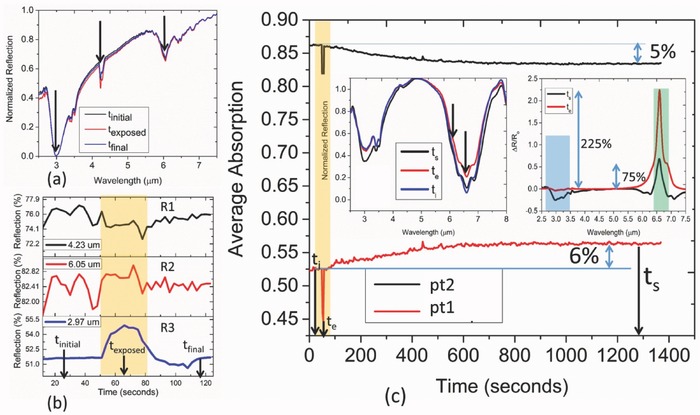
Characterization of 300 nm PEI film coated on a flat metal surface as exposed to a concentration of 1000 ppm followed by a flush with ambient CO_2_ gas at 75th second. The initial condition is maintained at ambient CO_2_ concentration, i.e., 600 ppm a). Broadband mid‐IR spectra of the three consecutive cases b). Time evolution of the signals at three distinct wavelengths of the spectra. Fast recovery of the PEI film is clearly observed at the bottom panel (R3) c). Dynamic response of metamaterial average absorption band (400 nm) around the two different wavelengths (pt1: 6 µm and pt2: 6.60 µm) as exposed to 1000 ppm CO_2_. Left inset: Broadband mid‐IR spectra at three instances: t_i_ (initial condition: blue solid line), t_e_ (exposure: red solid line), and t_s_ (steady state: black solid line). Right inset: Differential spectra at two instances: t_e_ and t_s_ indicating large switching contrast achievable for detecting gas concentration above safety range.

In this section, we further report the dynamic sensing of CO_2_ gas beyond safe‐level, ambient CO_2_ concentration and selectivity against a range of volatile organic components. The upper limit for safe‐level CO_2_ at the workplace is regarded to be 1000 ppm. For the detection of gas at high concentration, we exploit the diffusion controlled sorption of gas within the thin film. The time‐dependent characteristics of the large area (100 µm by 100 µm) PEI film on metal as exposed to 1000 ppm concentration followed by a flush of room emperature CO_2_ is shown in **Figure** **9**a. Characteristics at various important wavelenths (R1, R2, and R3) are shown in Figure 9b. Even though the CO_2_ desorption is an endothermic process (i.e., requires external heat), we clearly observe a fast recovery behavior at certain wavelength (R3) when flushed with ambient CO_2_ concentration at 75th second. This essentially indicates the weak chemical binding strength between the amine branches and excess CO_2_ molecule at concentration beyond the capacity of the thin film and opens up the possibility for the recovery of the hybrid sensor at room temperature. Here, we leverage the diffusion controlled behavior for sensing of high concentration CO_2_ using metmatierial absorber. As seen in Figure 9c, a steady state absorption difference (5–6%) can be achieved in around 10 min even when the sample is *pre‐exposed* to ambient CO_2_ concentration (600 ppm) and humidity (72%) over a prolonged period of time (24 h) at room temperature (25°). Note that the initial state of the sensor differs from that in Figure 2e as the condition prior to sensing is different. Interestingly, the dynamic intensity difference can transiently increase by 225% at the instance of gas injection due to the partial desorption and eventually settles down to 75% at steady state. The partial disorption appears to be mainly associated with the removal of the infrared chemical species at the upper surface of the material. The sharp decline in the time‐dependent average absorption collected at the two different wavelengths (pt1: 6 µm and pt2: 6.60 µm) plotted in Figure 8c also indicates this. The steady‐state difference observed implies the usefulness of the perfect absorption condition of metamaterial absorber for repeated gas sensing applications under ambient condition. The average absorption at pt2 steadily decreases from the initial value (86.50%) to a final value (82.5%) instead of increasing as the kinetically slow diffusion takes place. Average absorption is found to increase from 52% to 58% at pt1 on the other hand. However, at a lower concentration, average absorption at both the wavelengths shows the rising trend with increasing concentration (Figure 8d,e). A nonmonotonic trend is again observed at pt3 (Figure S9a, Supporting Information) implying the effect of spectral overlap within a given absorption bandwidth (400 nm). The rising trend at pt4 (Figure S9b, Supporting Information) also contradicts with the decreasing trend previously observed (Figure 8g). This indicates the sensing mechanism based on fast adsorption at the upper surface (**for low concentration CO_2_**) and slow diffusion of the chemisorbed CO_2_ in the bulk network (**for high concentration CO_2_**) can be unique from one another on the hybrid platform. The trend of the dynamic absorption, in fact, shows strong dependence on the operating wavelength and the corresponding bandwidth (Figure S10, Supporting Information). The proposed mechanism, therefore, offers multiple routes for resolving CO_2_ concentration in various ambient conditions. We believe, the low concentration characteristics will fit into emergency CO_2_ leak detection application in the refrigeration/storage system while the high concentration characteristics will serve for air quality monitoring at the industrial workplace.

**Figure 10:**
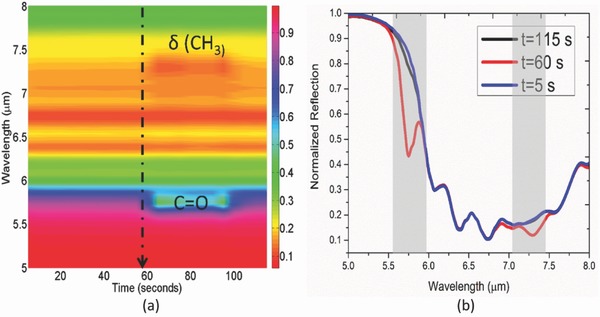
a) Wavelength versus time mapping of resonance profile of hybrid metamaterial absorber structure (C9) as exposed to representative volatile organic compound (acetone vapor) at the 58th second. The structure is pre‐exposed to 177 ppm CO_2_ before acetone exposure. Presence of transient vibrational modes of acetone is indicated accordingly. b) Extracted resonance profiles at three different instances. Transient interferences caused by the acetone molecule are marked the gray region in the spectra. Only the pt4 and pt5 spectral locations are affected due to the transient coupling with the δ CH_3_ finger print of volatile organic compound.

The selectivity experiment conducted on the hybrid sensor further shows the selectivity of the platform against a range of volatile organic compounds (**Figure** **10**a,b). Among the five spectral points under consideration, only pt4 and pt5 experience the transient effect of the volatile compound. Therefore, a careful selection of the operating wavelength can still enable the selective detection of CO_2_ in a mixed pollutant environment.

## 4 Conclusion

In summary, we demonstrate a novel solution for miniaturized gas sensor with ppm range of detection limit and high selectivity by coupling a smart, gas‐selective material with the metamaterial absorber platform. The hybrid metamaterial absorber‐based sensor further offers fast response time, large differential response for all‐optical monitoring, and low hysteresis and holds promise for direct integration with the existing NDIR system. As an added advantage, a dual mode sensing mechanism is demonstrated harnessing the full potential of the gas‐selective material. In particular, the infrared active functional groups of the gas‐selective polymer allow multiple unique sensing characteristics at low and high gas concentration as determined by the judicial selection of operating wavelength and spectral bandwidth. Saturation behavior of the thin film‐based sensor in continuous mode is expected to be furhter improved by (i) increasing the effective sensing area, (ii) engineering the gas‐selective layer for higher molecular weight, and (iii) strengthening light‐matter interaction by metamaterial absorber structure with nanogap supported field enhancement. The developed principle is expected to be extended further for the detection and sensing of various other greenhouse gases utilizing appropriate gas‐selective polymers. Finally, CMOS compatibility of the core absorber layer will be a great advantage for low‐cost implementation of the hybrid scheme in large‐scale gas sensing under the framework of the internet of things.

## Conflict of Interest

The authors declare no conflict of interest.

## Supporting information

Supplementary
