# Supplementary material for: Hybrid Metamaterial Absorber Platform for Sensing of CO2 Gas at Mid‐IR
Source: Adv Sci (Weinh). 2018 Feb 21;5(5):1700581. doi: 10.1002/advs.201700581 (PMC5978960; doi:10.1002/advs.201700581)
Supplement: Supplementary file 1 — Supplementary [file ADVS-5-1700581-s001.pdf]

Copyright WILEY-VCH Verlag GmbH & Co. KGaA, 69469 Weinheim, Germany, 2018.

ADVANCED SCIENCE
Open Access

Supporting Information

for *Adv. Sci.,* DOI: 10.1002/advs.201700581

Hybrid Metamaterial Absorber Platform for Sensing of CO_2 Gas at Mid-IR

*Dihan Hasan and Chengkuo Lee**

Supporting Information

Title

Hybrid Metamaterial Absorber Platform for Sensing of CO2 Gas at Mid-IR

Author list
Dihan Hasan, and Chengkuo Lee

E-mail: elelc@nus.edu.sg

Table of Content

Supplemental Section 1: Triboelectric charge based characterization of PEI membrane under various CO_2 conditions

Supplemental Section 2: Simulated effect of varying length of the nanostructure on the coupling with mid-infrared finger prints

Supplemental Section 3: Uniformity study of the hybrid absorber structure for sensing applications

Supplemental Section 4: Transient characteristics of thermal desorption

Supplemental Section 5: Transient characteristics of average absorption in the non-resonant regime

Supplemental Section 6: Dynamic response of the sensor toward high concentration CO_2 exposure

Supplemental Section 1: Triboelectric charge based characterization of PEI membrane under various CO_2 conditions

We begin with the characterization of CO_2 selective layer by looking at triboelectric charge generation expressed in terms of the open circuit voltage (VOC) illustrated in Figure 2 (left column). We obtain 300 nm thick film from 50% PEI (Mn ~60,000) diluted in DI water at 1:20 ratio at a spinning speed of 1000 rpm. A Keithley electrometer integrated with a moveable stage is deployed for the capture of VOC. The reference signal is obtained of the 4cm by 4 cm sample in Figure 2(a) consisting of bare PDMS membrane [1]. A peak open circuit voltage around 60 V is observed in Figure 2(b) for the given case that goes back to 0 v when the top ITO electrode touches the PDMS layer. The open circuit voltage drops to 20 V in the case of Figure 2(c) when the CO_2 free PEI layer is present on PDMS. Note that, PEI is positively charged

by nature. The presence of positive charge can offset the surface charge negativity (σ) of PDMS leading to the decrease of electric field defined as σ/ϵ in this case. We further observe the decrease of open circuit voltage when the PEI layer is exposed to ambient CO_2. The additional decrease can be attributed to the generation of complex ions upon exposure to CO_2.

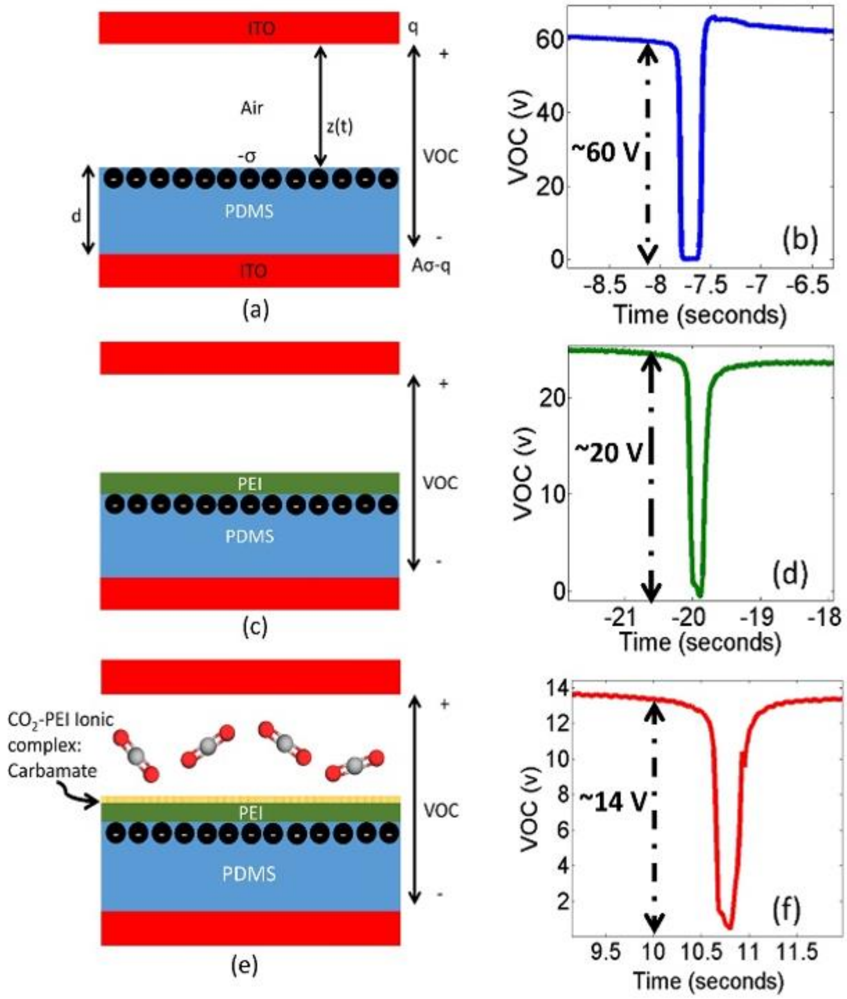

Figure S1: Triboelectric charge based characterization of PEI membrane. Open circuit voltage (VOC) between (a) ITO and reference PDMS (b) ITO and PDMS coated with 300 nm PEI layer baked at 100 deg. in N_2 environment for CO_2 desorption (c) ITO and PDMS coated with 1μm PEI after exposed to ambient CO_2 concentration (600 ppm)

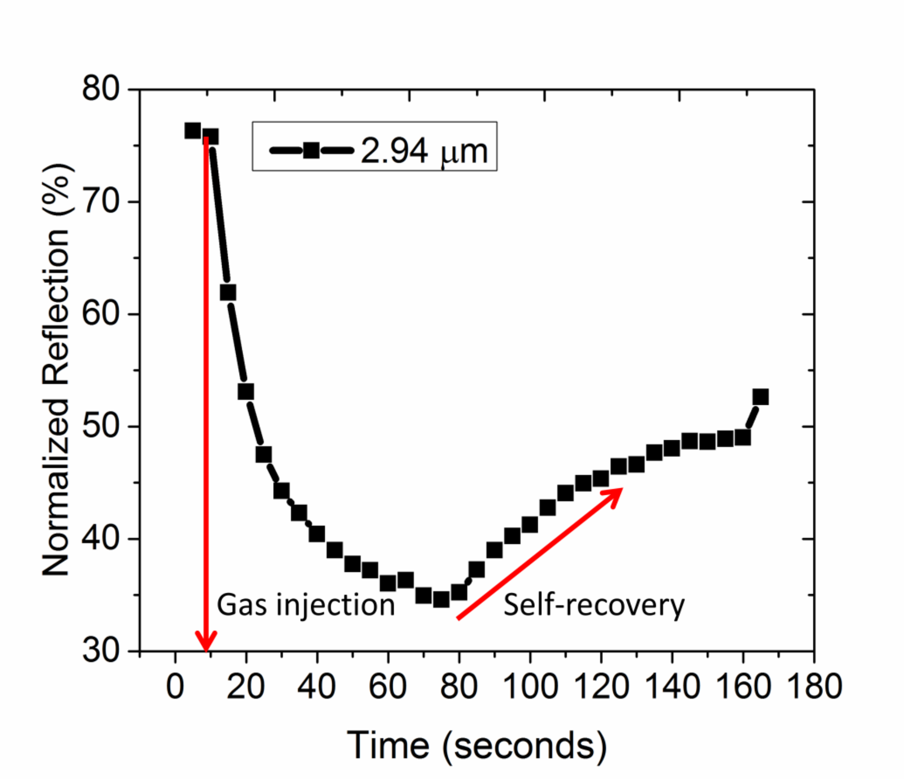

Figure S2: Self-recovery characteristics of the gas selective film at 2.94 μm (3401.36 cm^{-1})

[1] Lin, Z.-H.; Zhu, G.; Zhou, Y. S.; Yang, Y.; Bai, P.; Chen, J.; Wang, Z. L. A Self-Powered Triboelectric Nanosensor for Mercury Ion Detection. Angewandte Chemie International Edition 2013, 52, 5065–5069.

Supplemental Section 2: Simulated effect of varying length of the nanostructure on the coupling with mid-infrared finger prints

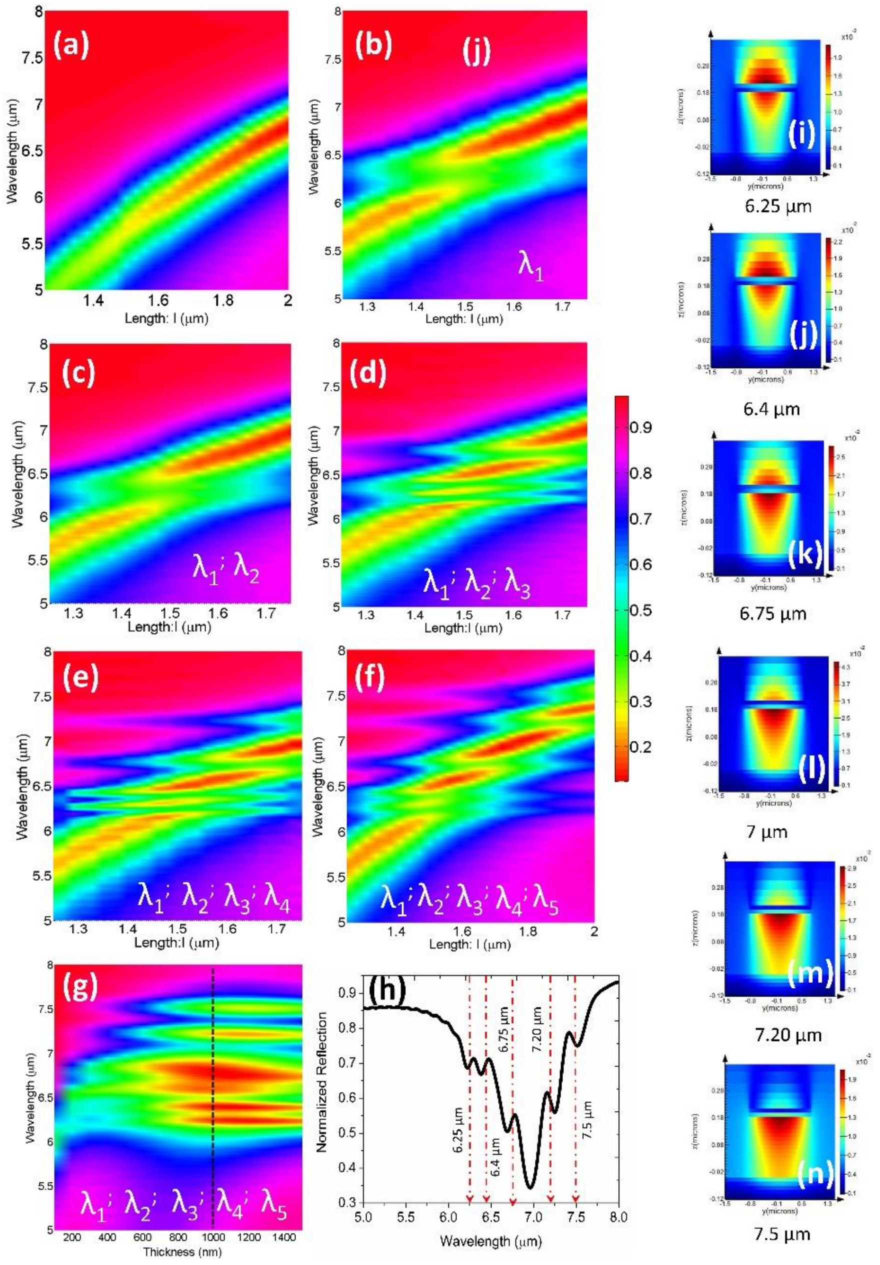

Figure S3: Wavelength versus length mapping for (a) representative simulation of hybrid absorber structure in the absence of any vibrational mode absorption (b) vibrational mode at λ_1= 6.25 µm (c) vibrational modes at λ_1= 6.25 µm and λ_2= 6.4 µm (d) vibrational modes at λ_1= 6.25 µm , λ_2= 6.4 µm and λ_3= 6.75 µm (e) vibrational modes at λ_1= 6.25 µm , λ_2= 6.4 µm , λ_3= 6.75 µm and λ_4= 7.20 µm (f) vibrational modes at λ_1= 6.25 µm , λ_2= 6.4 µm , λ_3= 6.75 µm, λ_4= 7.20 µm and λ_5= 7.5 µm. Clearly a broadening of effective absorption band is observed as more vibrational modes are coupled into the metamaterial resonance mode (g) Wavelength versus thickness mapping while the length is fixed at 1. 83 µm. Effective absorption reaches maximum at a thickness of 1000 nm (h) Extracted spectrum at length =1. 83 µm and thickness = 200nm. Magnetic field intensity distributions across XZ plane at (i) 6.25 µm (j) 6.4 µm (k) 6.75 µm (l) 7µm (m) 7.20 µm (n) 7.5 µm. The field distributions indicate the magnetic nature of the background metamaterial resonance with the potential for efficient light-to-heat conversion for further integration. The gas selective material is modeled by five Lorentz oscillators [1].

The Fano type resonances are defined by $\epsilon = \epsilon_o + \sum_i \frac{\epsilon_{lorentz}\omega_i^2}{\omega_i^2 - 2i\delta_o\omega - \omega^2}$ where $\epsilon_o =$ 2.2500. $\epsilon_{lorentz}$ and δ_o are fixed at 0.005 and 8e11 rad/s ,respectively [1]

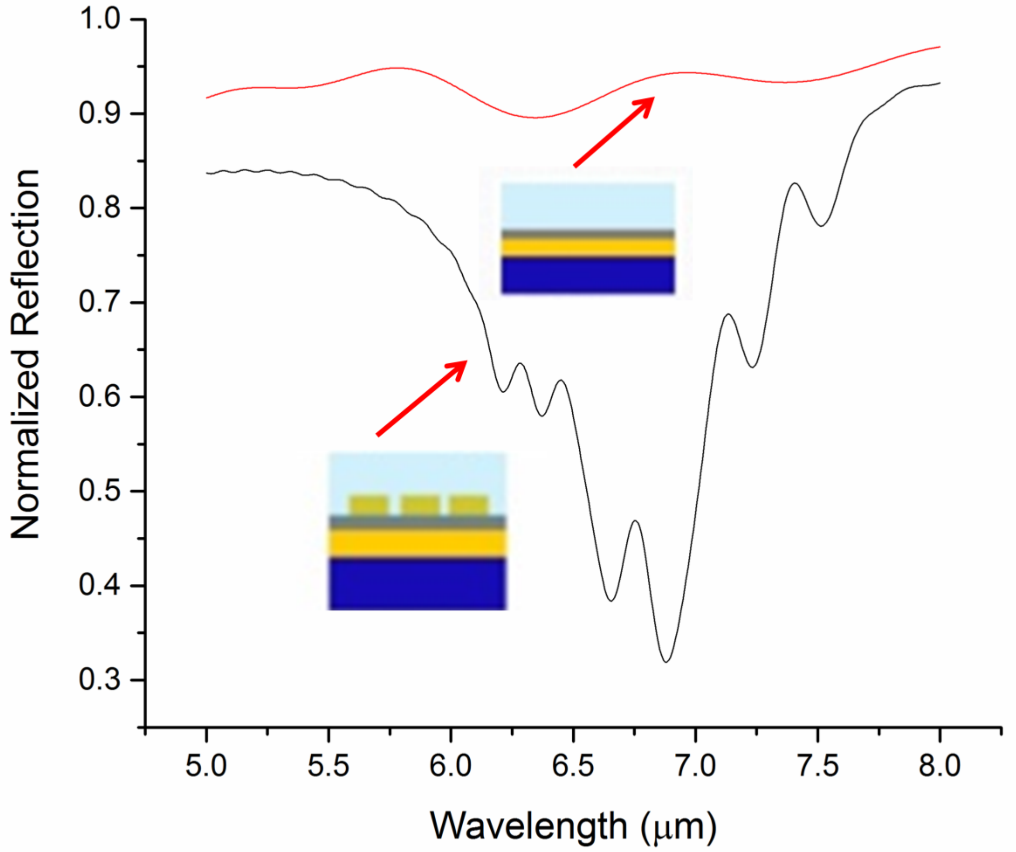

Figure S4 Simulated comparison of the effect of metamaterial absorption resonance on the coupling of the closely spaced material vibrational modes. In the absence of the properly designed metamaterial pattern, coupling into the vibrational modes is not strong enough to resolve one from the other

[1] Lahiri, B.; McMeekin, S. G.; De La Rue, R. M.; Johnson, N. P. Enhanced Fano Resonance of Organic Material Films Deposited on Arrays of Asymmetric Split-Ring Resonators (A-SRRs). *Optics Express* **2013**, *21*, 9343.

Supplemental Section 3: Uniformity study of the hybrid absorber structure for sensing applications

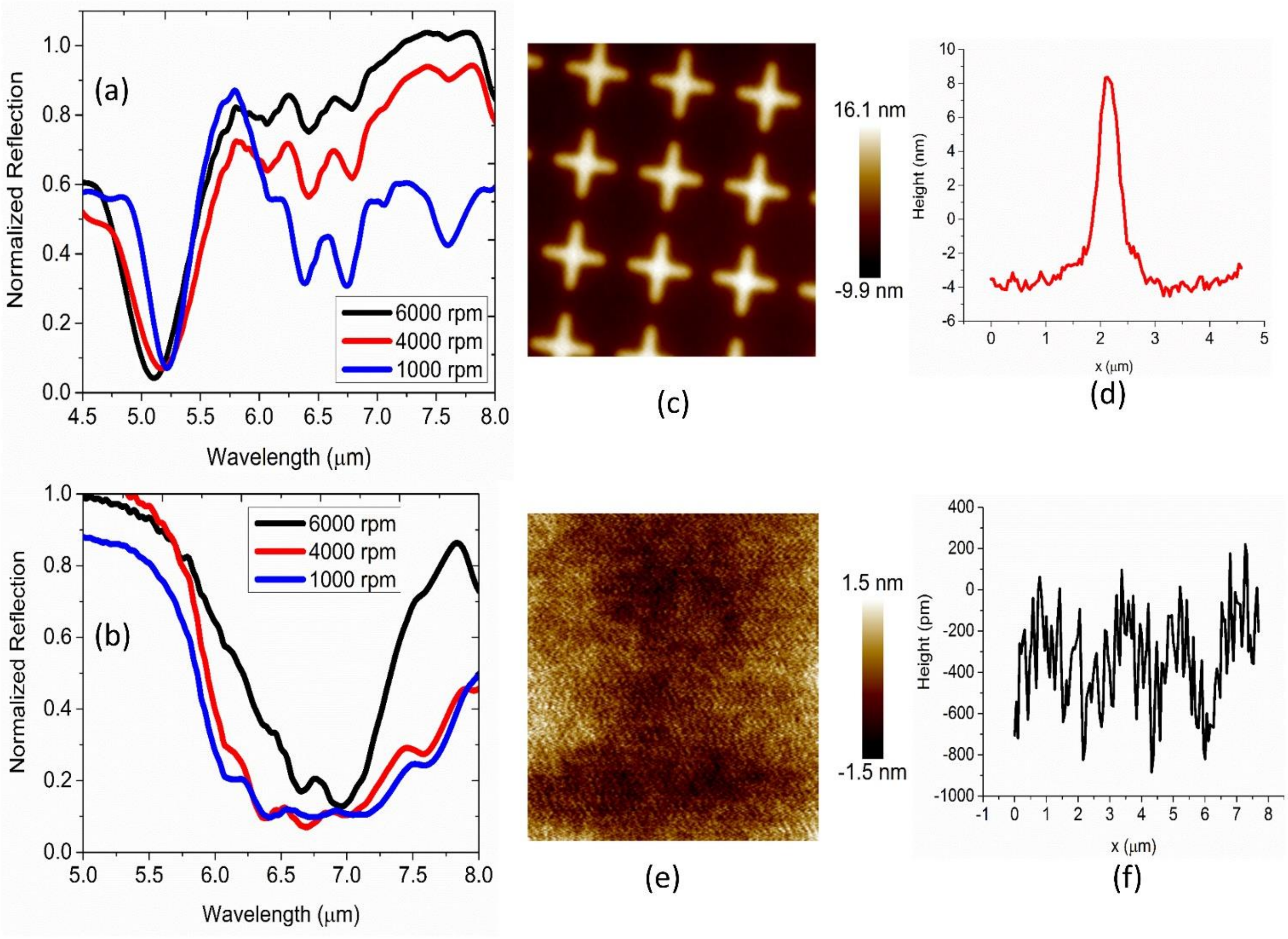

Figure S5: Effect of thickness variation observed by varying spinng speed (rpm) (a) C2 structure (b) C9 structure A clear broadening is observed in C9 as the thickness is increased while a consistent red shift of C2 resonance is observed in C2 (c) AFM imaging of C2 structure spin coated by PEI film at 6000 rpm (d) AFM height difference that shows the thickness obtained to be 125 nm (e) AFM imaging of C2 structure spin coated by PEI film at 1000 rpm (f) AFM imaging showing no surface topology

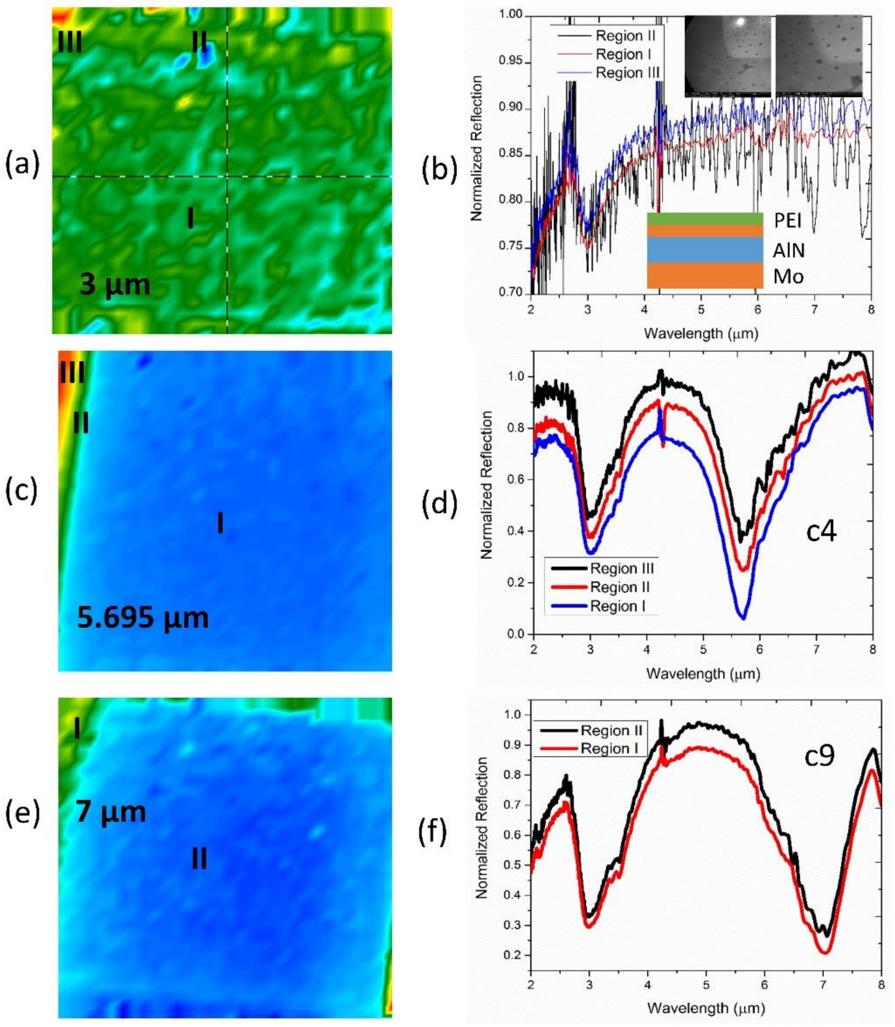

Figure S6: (a) Focal plane array (FPA) imaging of PEI film coated on continuous metal surface. The imaging wavelength is 3 µm (b) Corresponding mid-IR spectra extracted from three different regions as indicated. Inset: cracks observed in the PEI film coated on metal surface after several cycles of heat treatment. Such sub-wavelength cracks are found not to interfere with the characteristics of hybrid metamaterial device (c) FPA imaging of C4 resonance collected at 5.695 µm (d) Corresponding mid-IR spectra extracted from three different regions as indicated (e) FPA imaging of C9 resonance collected at 7 µm (d) Corresponding mid-IR spectra extracted from different regions as indicated

Supplemental Section 4: Transient characteristics of thermal desorption

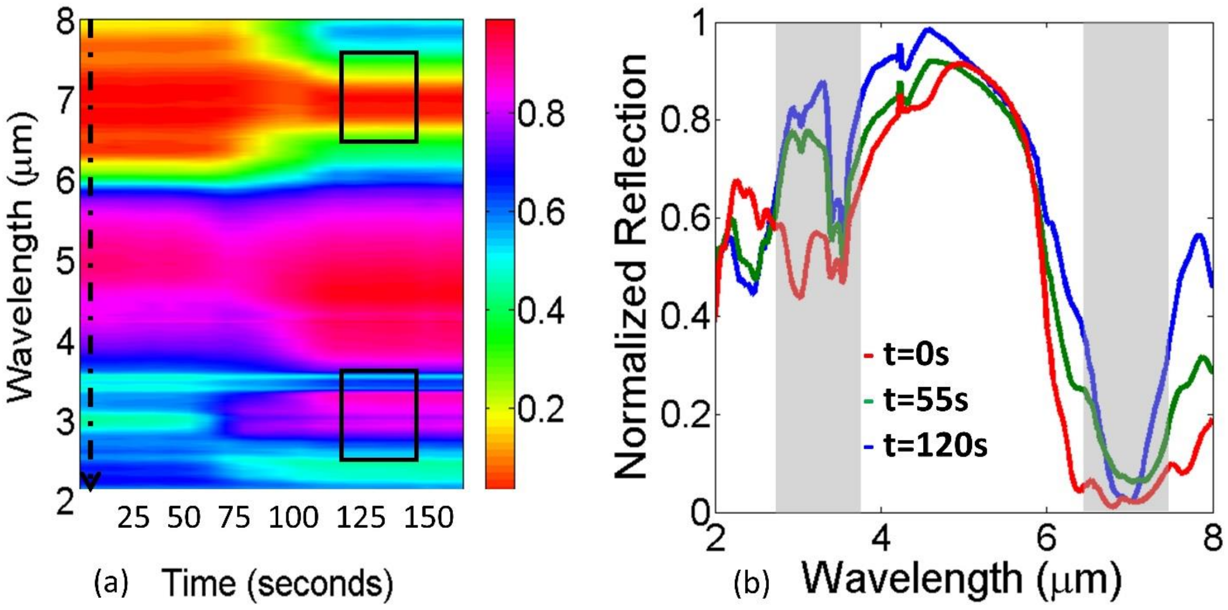

Figure S7 (a) Wavelength versus time mappin of thermal desorption. Annealing starts at the instance (5th second) indicated. Sensor starts to reset for next measurement within ~60s . The rectangles indicate the gas selective l regions of the spectral window in consideration of this work (b) Corresponding spectral profiles captured at three representive instances showing complete recovery of the sensor in 120s

Supplemental Section 5: Transient characteristics of average absorption in the non-resonant regime

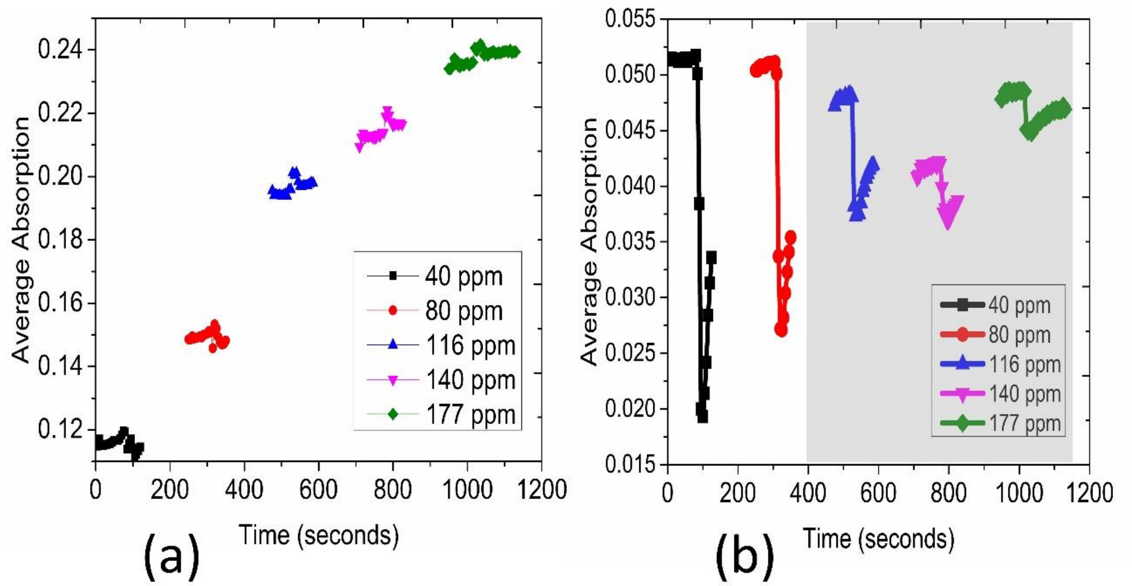

Figure S8 Dynamic behaviour of average absorption at two different wavelengths falling under non-resonant regime (a) 4.23 μm and 5.6 μm

Supplemental Section 6: Dynamic response of the sensor toward high concentration CO_2 exposure

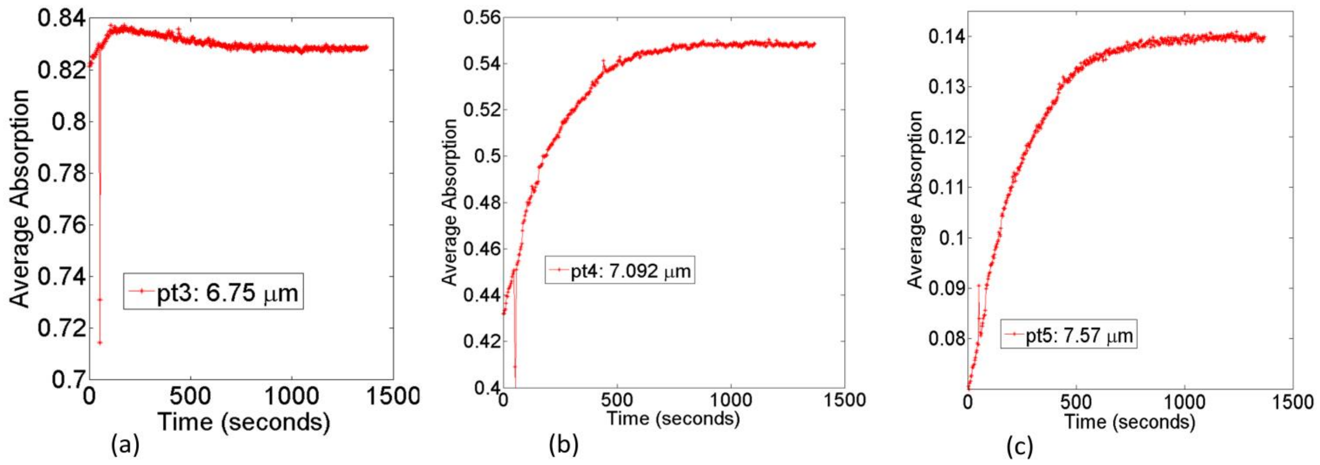

Figure S9 Dynamic response of metamaterial average absorption band (400 nm) around the three different wavelengths as exposed to 1000 ppm CO_2 (a)pt3 (b) pt4 (c) pt5

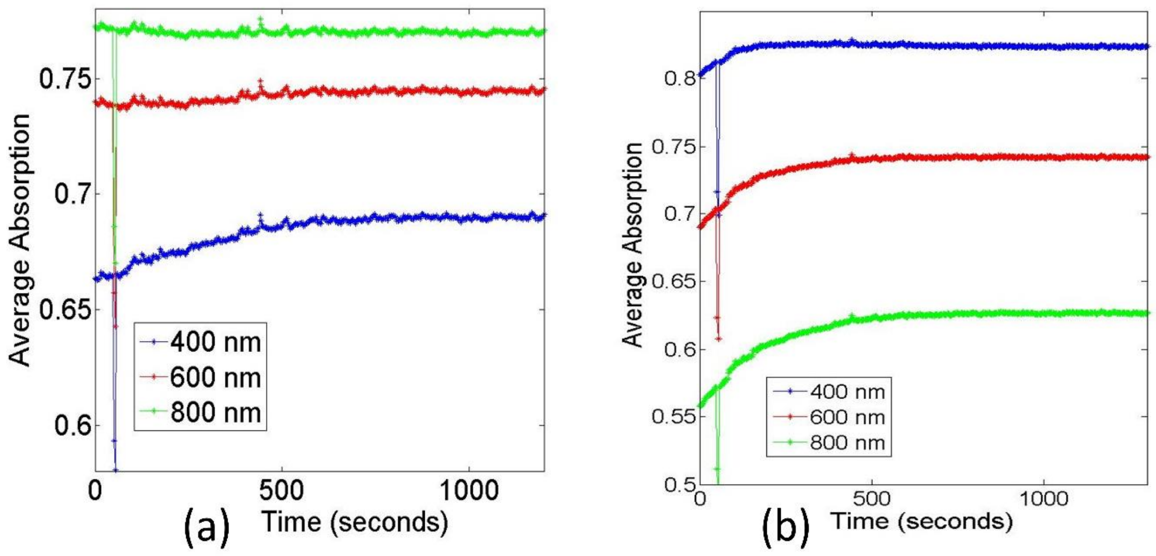

Figure S10 Effect of operating wavelength and bandwidth on the average absorption as the sensor is exposed to 1000 ppm CO_2 (a) pt1: 6.0 µm (b) pt3: 6.75 µm
